# Prenatal kisspeptin antagonist exposure prevents polycystic ovary syndrome development in prenatally-androgenized rats in adulthood: An experimental study

**DOI:** 10.18502/ijrm.v21i2.12801

**Published:** 2023-03-08

**Authors:** Elahe Sadeghian Bakhi, Nasim Hayati Roodbari, Morteza Anvari, Fahimeh Ramezani Tehrani

**Affiliations:** ^1^Department of Biology, School of Basic Science, Science and Research Branch, Islamic Azad University, Tehran, Iran.; ^2^Research and Clinical Center for Infertility, Yazd Reproductive Sciences Institute, Shahid Sadoughi University of Medical Sciences, Yazd, Iran.; ^3^Reproductive Endocrinology Research Center, Research Institute for Endocrine Sciences, Shahid Beheshti University of Medical Sciences, Tehran, Iran.

**Keywords:** Androgen, Kisspeptin antagonist, Polycystic ovary syndrome, Rat.

## Abstract

**Background:**

Increased levels of kisspeptin are associated with hypothalamus-pituitary-ovary axis dysfunction. It may lead to the development of polycystic ovary syndrome (PCOS).

**Objective:**

We aimed to investigate the effect of prenatal kisspeptin antagonist exposure on the development of PCOS in prenatally androgenized rats in adulthood.

**Materials and Methods:**

In this experimental study, pregnant rats were injected with free testosterone (T, 5 mg/day) or T+P271 (kisspeptin antagonist) on the 20 ${}^{\text{th}}$ day of the pregnancy period (n = 5 in each group), while rats in the control group received solvent. Female offspring were examined in terms of anogenital distance (AGD), anovaginal distance (AVD), vaginal opening, serum total testosterone (TT) levels, ovarian follicles, and the regularity of estrous cycles in adulthood. AGD and AVD were measured using a vernier caliper. TT levels were measured using the enzyme-linked immunosorbent assay method. Ovaries were fixed in 10% formalin, tissue processing was done by a standard protocol, and then ovaries embedded in paraffin. 5 μm-thickness ovarian sections mounted on a glass slide, deparaffinized, and stained using Harris's Hematoxylin and Eosin Y.

**Results:**

AGD, AVD (p < 0.001), TT levels (p = 0.02), and the numbers of preantral and antral follicles (p < 0.001) in the ovaries were significantly decreased in prenatally T-P271-exposed rats compared to prenatally T-exposed rats. The age of vaginal opening was early in T-P271-exposed rats compared to prenatally T-exposed rats (p < 0.001). The number of corpora lutea was significantly increased in T-P271-exposed rats (p < 0.001). No cystic follicles were observed in the ovaries of prenatally T-P271-exposed rats. Prenatally T-P271-exposed rats had regular estrous cycles compared to prenatally T-exposed rats.

**Conclusion:**

Prenatal exposure to kisspeptin antagonist can prevent PCOS development in prenatally androgenized rats in adulthood.

## 1. Introduction

Polycystic ovary syndrome (PCOS), one of the most common endocrine disorders, affects 5-20% of reproductive-age women worldwide (1, 2). PCOS is associated with reproductive, metabolic, and public health disorders such as luteinizing hormone (LH) hypersecretion, hyperandrogenism (clinical and/or biochemical), ovarian cysts, oligo/anovulation, obesity, insulin resistance, and physical and emotional morbidities in those affected (3, 4).

Besides genetic factors, environmental factors, and epigenetic changes during fetal life, can affect the development of PCOS. Emerging evidence suggests that a hyperandrogenic intrauterine environment plays a central role in developing PCOS in adult life (5, 6). Exposure to high levels of androgens during critical periods of development (prenatal or early postnatal) can be associated with increased activity of gonadotropin-releasing hormone (GnRH) neurons and LH hypersecretion; leading to androgen excess as one of the main endocrine abnormalities in PCOS subjects (7, 8).

Kisspeptin, a recently discovered neuropeptide acts upstream of GnRH neurons and is the key regulator of the hypothalamic-pituitary-ovary (HPO) axis. The kisspeptin neural system plays an important role in the maturation and function of the reproductive system (9, 10).

A previous study reported that LH levels were directly related to kisspeptin levels (11). Women with PCOS have also had high levels of kisspeptin (12). PCOS can be described as the disturbed organization of the hypothalamic kisspeptin system, possibly due to exposure to abnormal levels of sex steroid hormones, such as androgens, during early life (12, 13). Previous studies have shown that kisspeptin mRNA levels and the number of kisspeptin neurons in the arcuate nucleus region of the hypothalamus were elevated in animal models of PCOS (14, 15). kisspeptin is considered as one of the activator factors for GnRH neurons, therefore the increasing in the number of kisspeptin-producing cells or the levels of kisspeptin mRNA could be a potential cause of increased GnRH neuron activity and LH secretion leading to PCOS development (16, 17). It has been shown that kisspeptin knockout in healthy mice causes LH pulse disruption, which is followed by irregular estrous cycles and defects in ovarian folliculogenesis (18).

Despite the prevalence of PCOS and its effects on health, there is no definitive curative option for this syndrome, and all the available treatment modalities relieve symptoms (19, 20). Due to the assumed regulatory role of kisspeptin on the HPO axis, the use of kisspeptin antagonist may be an appropriate therapeutic method for hormonal disorders, especially GnRH/LH pulse frequency in PCOS subjects (12, 13).

In the present study, we aimed to investigate the effects of prenatal exposure to a single dose of kisspeptin antagonist (P271) on the serum total testosterone (TT) levels, ovarian tissue, and the regularity of estrous cycles in prenatally androgenized rats (rat model of PCOS), in adulthood.

## 2. Materials and Methods

### Study design

This experimental study was conducted in 2018-2019 in the biotechnology department of Yazd Research and Clinical Center for Infertility, Yazd, Iran. In this study, the inclusion criteria were PCOS model.

### Animals 

Twenty female Wistar rats, weighing 170-190 gr, 75-85 days of age, were obtained from the animal center of Shahid Sadoughi University of Medical Sciences (Yazd, Iran). One pair of male and female rats were housed under standard conditions (12 hr light/dark cycle, temperature 22 ± 3 C, relative humidity of 45-55%, with free access to food and water ad libitum) in a polypropylene cage (43 cm × 30 cm × 15 cm) for 24 hr. After mating, observing the vaginal plug was considered the first day of pregnancy.

### Inducing a rat model of PCOS and exposure to kisspeptin antagonist (P271) 

We used the same method previously published to induce a rat model of PCOS. In summary, in the experimental group, pregnant rats (n = 10) received 5 mg of free testosterone (T) (T1500; Sigma, Steinheim, Germany) dissolved in a 500 μl cocktail containing sesame oil (S3547; Sigma, Steinheim, Germany) and benzyl benzoate (B6630; Sigma, Steinheim, Germany) in a 4:1 ratio by subcutaneous injection (s.c.) on the 20 ${}^{\text{th}}$ day of pregnancy period. On the other hand, in the control group (vehicle), pregnant rats (n = 10) on the 20 ${}^{\text{th}}$ day of their pregnancy received only 500 μl of solvent simultaneously with s.c. injection (20).

The experimental group was randomly divided into 2 groups. Group 1, pregnant rats (n = 5) were treated by intraperitoneal (i.p.) injection of 1 μl P271 (Peptide 271, EZBiolab, Carmel, CA, USA), 3 hr after testosterone injection, while group 2, pregnant rats (n = 5), received only 200 µl of phosphate-buffered saline (PBS), 3 hr after testosterone injection (21). Female offspring of androgenized rats (group 2) were considered as the prenatally-androgenized rat model of PCOS (20).

In addition, the control group were also randomly divided into 2 groups. Group 3, pregnant rats (n = 5) were i.p. injected by 1 μl P271, 3 hr after solvent injection, while group 4 (n = 5) received only PBS, 3 hr after solvent injection (21).

**Figure 1:**
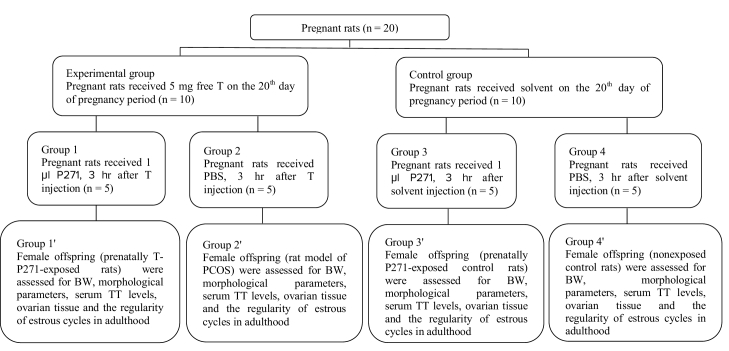
Flowchart of study, T: Testosterone, Solvent: Sesame oil and benzyl benzoate, P271: Kisspeptin antagonist, PBS: Phosphate-buffered saline, BW: Body weight, TT: Total testosterone, PCOS: Polycystic ovary syndrome.

After weaning, female offspring of 4 groups, including prenatally T-P271-exposed rats (group 1*'*), non-exposed PCOS rats (group 2*'*), prenatally P271-exposed control rats (group 3*'*), and non-exposed control rats (group 4*'*) were kept in groups of 4 per cage with free access to food and water. All female offspring were assessed in terms of body weight, morphological parameters, serum TT levels, ovarian tissue, and the regularity of estrous cycles in later life (in adulthood). The selection process of rats is presented in figure 1.

### Determination of body weights (BWs) 

The BWs of the female offspring of all study groups (n = 16 in each group) were measured at birth and 15, 30, 45, 60, and 75 days of age by a digital scale (Japan 2J-V1000AMax1200 gr, accuracy 0.01 gr).

### Measurement of anogenital distance (AGD) and anovaginal distance (AVD) and examination of the vaginal opening (VO)

The AGD (the distance (mm) between the cranial edge of the anus and the base of the phallus) and the AVD (the distance (mm) between the anterior edge of the anus and the posterior edge of the vaginal orifice) were measured at 15, 30, 45, 60, and 75 days of age using a vernier caliper, for all offspring. VO, as one of the physiological components of sexual maturation in female rats, was checked during 30-45 days of age, and the day of VO was recorded for each female offspring (n = 16 in each group) (22).

### Evaluation of estrous cycle 

Microscopic observations of vaginal smears were performed to assess our study rats' regularity or irregularity of the estrous cycles. Vaginal smears were collected between 12:00-4:00 PM for 15 consecutive days for all female offspring (age 60-75 days) (n = 16 in each group). Air-dried vaginal smears, collected on glass slides, were stained with crystal violet and examined by light microscopy (100x magnification). Estrous cycle phases were determined based on the predominant specific cells including round nucleated epithelial cells, cornified squamous cells, and leucocytes. The procedure for collecting vaginal samples has been explained in the previous studies (20, 23).

### Blood collection

At the estrus phase of the sexual cycle, adult female offspring (85-95 days of age, n = 8 in each group) were anesthetized with i.p. injection of a mixture of 50 mg/kg of 10% ketamine and 10 mg/kg of 2% xylazine (Alfasan, Woerden, Holland). After deep anesthesia, blood samples were taken from the heart. Blood samples were centrifuged at 6000 g for 5 min at 4 C. The sera were stored at -80 C for subsequent measurement of TT levels (20).

### Measurement of TT levels

Serum TT levels were measured by an electrochemiluminescence immunoassay kit (ECLIA,cobasⓇ, Roche Elecsys e 411, Switzerland). The sensitivity of the kit was 0.02-15.0 ng/ml. Intra-assay coefficients of variation for TT were < 10%.

### Ovarian histological examination

After the blood collection, rats were killed by heart incision, and the ovaries were immediately removed from the body cavity and prepared for histological studies using the method described by Baali et al. (24), with some modifications. In brief, the connective tissue of the ovaries were removed, and then their weight was measured with a digital scale and placed in 10% formalin at room temperature for 3-5 days for fixation. Processed according to a standard protocol and embedded in paraffin. The ovarian tissues were dehydrated by increasing the ethanol concentration (70%-100%), cleared with xylene, and paraffinized in the automatic tissue processor machine for a certain period. A 5-μm-thick serially sectioned ovarian were mounted on glass slides, and deparaffinized by xylene, hydrated by an ethanol series (100%, 90%, 80%, 70%, and 50%) and distilled water, stained using Harris hematoxylin and eosin (H&E staining). Ovarian histological sections were examined under light microscopy (40X and 100X magnifications). 5 representative sections in each ovary at least 30 μm apart were examined. The numbers of preantral, antral, preovulatory, and cystic follicles, as well as the number of corpora lutea were determined. For more accuracy the numbers of follicles and corpora lutea were determined by 2 experts.

### Ethical considerations

In this study, all experimental protocols were approved by the local Ethics Committee Islamic Azad University, Yazd Branch, Yazd, Iran (Code: IR.IAU.YAZD.REC.1397.004). All ethical protocols for working with laboratory animals were observed in this study.

### Statistical analysis

Continuous variables were checked for normality based on the one-sample Kolmogorov-Smirnov test. Data were presented as median with inter-quartile range (IQ25-75) for variables with skewed distribution. The Kruskal-Wallis H test followed by post hoc Dunn's test was applied to compare variables with skewed distribution. The generalized estimating equation (GEE) method was used to estimate the coefficient of interest in a generalized linear model, where each phase was considered as a repeated measure for 4 groups. The independent working correlation matrix was assumed according to the study's design. Compared to generalized linear model methods, the GEE approach is more consistent when data has not fulfilled the normality assumption. To assess the effect of P271 treatment on the outcomes of interest (BW, AGD, AVD), the model was adjusted for group (PCOS and non-PCOS) and phase studies. We designed a model in which the main effects of the P271 treatment, PCOS, and phase of the study were estimated. In addition, 2- and 3-way effect modifier variables (interaction effect) of P271 treatment phase, PCOS phase, and P271 treatment PCOS phase were estimated to check the effects of P271 treatment, adjusted by PCOS and phase during follow-up on the outcome of interest.

All statistical tests were performed by SPSS software (V.24 /00 SPSS, Inc., Chicago). P < 0.05 was considered statistically significant.

## 3. Results

### BWs

**Figure 2:**
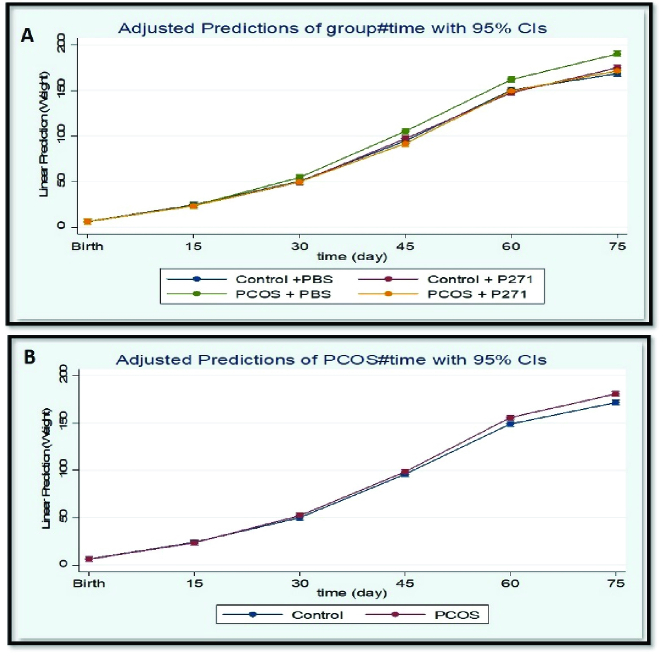
(A-B) The body weight (BW) profiles of study rats at birth, 15, 30, 45, 60, and 75 days of age (n = 16 in each group). A) Comparison of BWs between groups 1*'*, 2 *${}^{{}^{'}}$ *, 3 *${}^{{}^{'}}$* and 4 *${}^{{}^{'}}$ *, B) Comparison of BWs between PCOS and non-PCOS (control) groups. The results are based on the generalized estimating equation (GEE) method. PCOS + P271: Prenatally T-P271-exposed rats (Group 1*'*), PCOS + PBS: Nonexposed polycystic ovary syndrome (PCOS), rats (Group 2 *${}^{{}^{'}}$ *), Control + P271: Prenatally P271-exposed control rats (Group 3 *${}^{{}^{'}}$ *), Control + PBS: Nonexposed control rats (Group 4 *${}^{{}^{'}}$ *), P271: Kisspeptin antagonist, T: Testosterone, PBS: Phosphate-buffered saline.

A comparison of the BWs of prenatally T-P271-exposed and nonexposed PCOS rats compared to controls at different ages is presented in figure 2. No significant differences were observed in the BWs of female offspring of all 4 study groups at birth, 15, and 30 days of age (p > 0.05). While BWs in group 1*' *were significantly lower than compared togroup 2*'* at 45, 60, and 75 days of age (p < 0.05), approximately reaching these values that were observed in control rats (groups 3*' *and 4*'*) (Figure 2).

### AGD and AVD

**Figure 3:**
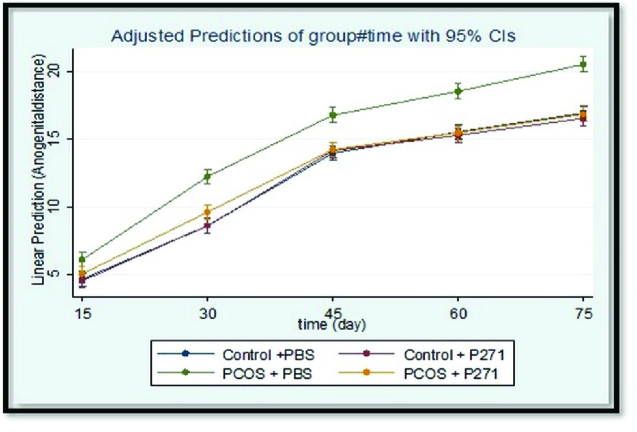
Comparison of anogenital distance (AGD) in groups 1*'*, 2 *${}^{{}^{'}}$ *, 3 *${}^{{}^{'}}$ *, and 4 *${}^{{}^{'}}$ *, at 15, 30, 45, 60, and 75 days of age. The results are based on the generalized estimating equation (GEE) method. (n = 16 in each group). PCOS + P271: Prenatally T-P271-exposed rats (Group 1*'*), PCOS + PBS: Nonexposed polycystic ovary syndrome (PCOS) rats (Group 2 *${}^{{}^{'}}$ *), Control + P271: Prenatally P271-exposed control rats (Group 3 *${}^{{}^{'}}$ *), Control + PBS: Nonexposed control rats (Group 4 *${}^{{}^{'}}$ *), P271: Kisspeptin antagonist, T: Testosterone, PBS: Phosphate-buffered saline.

**Figure 4:**
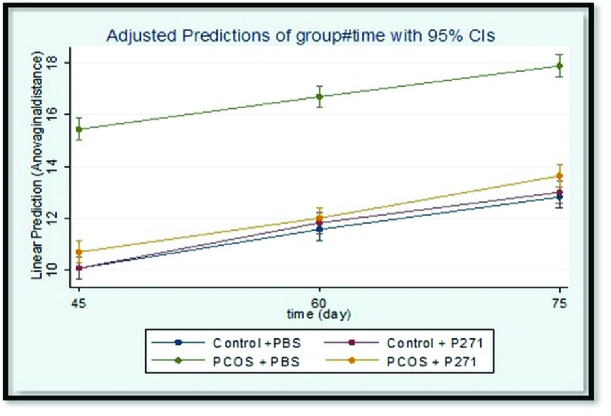
Comparison of anovaginal distance (AVD) in groups 1*'*, 2 *${}^{{}^{'}}$ *, 3 *${}^{{}^{'}}$ *, and 4 *${}^{{}^{'}}$ *, at 45, 60, and 75 days of age. The results are based on the generalized estimating equation (GEE) method. (n = 16 in each group). PCOS + P271: Prenatally T-P271-exposed rats (Group 1*'*), PCOS + PBS: Nonexposed polycystic ovary syndrome (PCOS) rats (Group 2 *${}^{{}^{'}}$ *), Control + P271: Prenatally P271-exposed control rats (Group 3 *${}^{{}^{'}}$ *), Control + PBS: Nonexposed control rats (Group 4 *${}^{{}^{'}}$ *), P271: Kisspeptin antagonist, T: Testosterone, PBS: Phosphate-buffered saline.

Figures 3 and 4 show the results of AGD and AVD measurements in groups 1*'*, 2*'*, 3*'*, and 4*'*. There were statistically significant lower than in AGD and AVD in group 1*'* compared to group 2*'* (p < 0.001). Generally, in group 1*'*, AGD and AVD were similar to the control groups (groups 3*'* and 4*'*).

### VO

**Figure 5:**
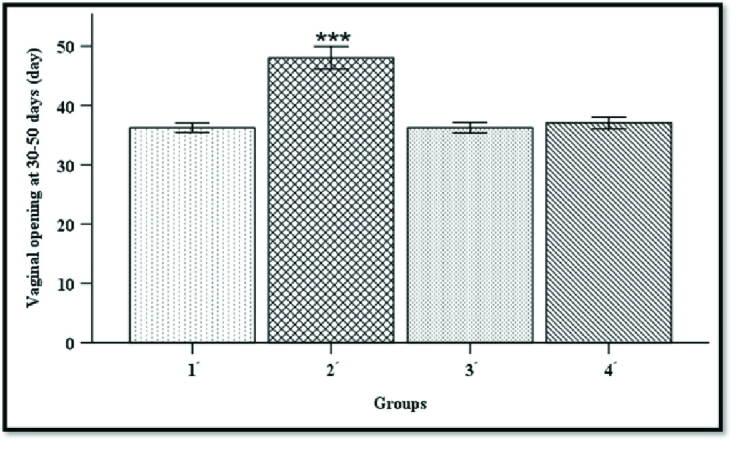
Comparison of the vaginal opening in groups 1*'*, 2 *${}^{{}^{'}}$ *, 3 *${}^{{}^{'}}$ *, and 4 *${}^{{}^{'}}$* at 30-45 days of age. Kruskal-Wallis H test was used to compare the results between groups (n = 16 in each group). ${}^{***}$ P < 0.001, Group 1*'*: Prenatally T-P271-exposed rats, Group 2 *${}^{{}^{'}}$ *: Nonexposed polycystic ovary syndrome (PCOS) rats, Group 3 *${}^{{}^{'}}$ *: Prenatally P271-exposed control rats, Group 4 *${}^{{}^{'}}$ *: Nonexposed control rats, P271: Kisspeptin antagonist, T: Testosterone.

Timing of VO, as a marker of puberty onset, was determined for all rats during 30-45 days of age. Based on our records, the age of VO was earlier in T-P271-exposed rats compared to prenatally T-exposed rats (rat model of PCOS). The timing of VO was longer in group 2*'* compared to rats in groups 1*'*, 3*',* and 4*'* (Figure 5) (p > 0.001).

### Estrous cycle

Observation of vaginal smears daily for 15 consecutive days demonstrated that rats in group 1*'*, had regular estrous cycles similar to those observed in control rats (groups 3' and 4'), while estrous cycles were irregular in rats of group 2'.

### TT levels

A significant reduction in the serum TT levels was observed in rats of group 1*'* compared to the rats of group 2*'* (p = 0.02), and the levels of TT in group 1*'* reached to the TT levels in controls (3*'* and 4*'*). Median and IQ25-75 in study's groups (1*'*, 2*',* 3*'* and 4*'*) were 0.02 (0.02-0.02), 0.02 (0.02-3.87), 0.02 (0.02-0.02), 0.02 (0.02-0.02), respectively (p = 0/02).

### Ovarian weight and morphology

No significant differences were observed in the ovarian weights of rats in 4 groups (1*'*, 2*'*, 3*'*, and 4*'*) (p = 0.4) (Table I).

The results of ovarian morphology are presented in table I. An increase was observed in the numbers of preantral and antral follicles ingroup 2 *${}^{{}^{'}}$* compared to 1*'*, 3*'*, and 4*'* groups in adulthood. Also, the number of preovulatory follicles and corpora lutea significantly lower than in rats of group 2 *${}^{{}^{'}}$ *. On the other hand, the number of follicles and corpora lutea in group 1*'* was similar to controls (groups 3*'* and 4*'*) (Table I) (p < 0.001).

**Table 1:** Number of preantral, antral, preovulatory follicles, and corpora lutea and ovarian weight in study's groups (82-90 days of age)


	**Groups**				
**Variable**	**1** ***'***	**2** ***'***	**3** ***'***	**4** ***'***	**P-value**
	**Preantral follicles**	10.00 (9.00-10.00)	13.00 (12.25-13.75) ${}^{***}$	9.00 (8.25-10.00)	9.50 (8.25-10.00)	< 0.001
**Antral follicles**	10.00 (9.00-10.75)	12.00 (11.25-13.75) ${}^{***}$	9.00 (8.25-9.75)	8.50 (7.25-9.00)	< 0.001
**Corpora lutea**	10.00 (10.00-10.75)	6.00 (4.25-6.75) ${}^{***}$	10.50 (9.25-11.00)	10.00 (9.25-10.00)	< 0.001
**Preovulatory follicles**	7.00 (6.00-7.75)	1.50 (1.00-2.00) ${}^{***}$	6.50 (5.00-8.75)	8.00 (7.00-9.00)	< 0.001
**Ovary weight**	0.06 (0.05-0.06)	0.06 (0.05-0.07)	0.05 (0.04-0.06)	0.05 (0.05-0.06)	0.4
Values are expressed as median and interquartile intervals (Q1-Q3). Kruskal-Wallis H test was used to compare the results between groups (n = 8 in each group), ${}^{***}$ P < 0.001, Group 1 * ${}^{{}^{'}}$ *: Prenatally T-P271-exposed rats, Group 2 * ${}^{{}^{'}}$ *: Nonexposed polycystic ovary syndrome (PCOS) rats, Group 3 * ${}^{{}^{'}}$ *: Prenatally P271-exposed control rats, Group 4 * ${}^{{}^{'}}$ *: Nonexposed control rats					

## 4. Discussion

In the present study, we found that prenatally-androgenized rats exposed to a single dose of P271 during fetal life (embryonic day 20) exhibit normal ovarian tissue in terms of folliculogenesis and ovulation, regular estrous cycles, and normal serum levels of TT in adulthood. Therefore, it seems that prenatal exposure to kisspeptin antagonist can prevent the development and appearance of PCOS in adult life, despite the exposure to androgens during fetal life.

Based on the evidence, a hyperandrogenic intrauterine environment plays a central role in developing PCOS in adulthood (25). Previous studies have shown that exposure to high levels of androgens during the critical periods of development (prenatal or early postnatal life); results in an increase in the GnRH surge-generating system and increased LH levels (7, 8); consequently, androgen excess that is one of the main endocrine abnormalities in PCOS subjects (20, 21). The underlying mechanisms that link the hyperandrogenic state with disturbances in GnRH pulse generator activity in the hypothalamus are incompletely understood; however, several assumed pathways have been reported so far. In a study conducted on female sheep exposed to androgens during the critical periods of development, a reduction in synaptic contact with GnRH neurons was observed (26); indicating changes in synaptic connectivity following exposure to androgens; leading to alterations in GnRH pulsation. Another study suggested that androgen receptor activation may cause changes in the movement of GABA-releasing neurons to GnRH neurons (27). Moreover, exposure to the supraphysiologic doses of androgens during fetal life may lead to the desensitization of GnRH neurons to the negative feedback of sex steroids (28). Reduced negative feedback of sex steroids has been reported in prenatally androgen-exposed female monkeys (29) and in women with PCOS (30). It may lead to increased pulsatile LH secretion and, subsequently, androgen excess. Furthermore, it was suggested that prenatal exposure to androgens may affect gonadotropic sensitivity to the GnRH stimulation (31).

Alterations in follicles number (increased numbers of preantral and antral follicles and a decrease in preovulatory follicles) in the ovary and the appearance of cystic follicles in our prenatally-androgenized rat model of PCOS are similar to the previous studies findings (20, 32). Generally, as discussed above, androgen overexposure during fetal life could alter the structure and function of the HPO axis in females. Disrupted folliculogenesis can be due to some possible mechanisms such as abnormality in the secretory function of the granulosa cells and excessive secretion of estrogens, decreased sensitivity of ovarian follicles to follicle-stimulating hormone, and an increase in the secretion of LH and testosterone leading to increased activity of cyclic adenosine monophosphate. Additionally, following an increase in the levels of androgen, intraovarian hydroxysteroid 11-beta dehydrogenase 1 activity is inhibited, which causes the formation of ovarian cysts as reported in women with PCOS (33).

In the present study, PCOS rats showed an irregular estrous cycle, a finding in agreement with previous studies (20, 34). This could be due to the irregularities and changes that occurred in the hypothalamic-pituitary-gonadal axis following prenatal exposure to androgens (35).

AGD is an anthropometric biomarker of the androgenic environment during the development of the reproductive system in fetal life and reveals reproductive health (36, 37). In the present study, AGD and AVD were increased in the prenatally-androgenized rat model of PCOS, indicating a male-like morphology, as reported in the previous study (20). This may be due to the presence and increase of androgen receptors before birth and their sensitivity to exogenous androgen before the final development of the reproductive tract.

In the present study, in agreement with previous studies, a significant increase in body weight of the rat model of PCOS compared to other groups was observed (20, 38). The relationship between obesity and PCOS has previously been reported (39). This increase may be due to increased insulin resistance in the PCOS group, which is due to decreased insulin binding to its receptor or defective in receptor autophosphorylation due to insulin receptor mutation (40).

Kisspeptin, a hypothalamic peptide encoded by the * kiss1* gene (41) as a regulator of the HPO axis, plays an important role in the onset of puberty and the maintenance of reproductive function (9, 15). It has been reported that kisspeptin through binding to its receptor controls GnRH secretion. Therefore, kisspeptin can involve in the pathophysiology of the HPO axis. A previous study proposed that exposure to elevated levels of androgens during early life can negatively affect the hypothalamic-kisspeptin system (13). Subsequently, it may lead to the development of PCOS. Studies in animals and humans have reported increased hypothalamic expression of kisspeptin and GnRH in PCOS conditions (12, 13, 42).

An experimental study indicated that kisspeptin antagonist decreases LH pulse frequency and amplitude (43). As a result, kisspeptin antagonist through deceasing in the activity of GnRH neurons may improve GnRH/LH pulse frequency in PCOS subjects (12, 21). In line with this evidence, our study results revealed that prenatal exposure to kisspeptin antagonist during the development of the HPO axis can prevent the appearance of PCOS phenotype (irregular sexual cycles, androgen excess, ovulation dysfunction, and ovarian cysts) in adulthood, despite exposure to androgens during fetal life. In our previous study conducted on prenatally-androgenized rats, decreases in GnRH mRNA expression, levels of sex steroid hormone, and gonadotropins were observed in prenatally-kisspeptin antagonist-exposed rats. Additionally, in agreement with a previous study, in the present study, kisspeptin-antagonist-treated rats showed regular estrous cycles, probably due to the inhibition of increased gonadotropin secretion and the inhibition of neuron GnRH activity following exposure to kisspeptin antagonist (21).

## 5. Conclusion

Prenatal exposure to kisspeptin antagonist can prevent PCOS development in adult life, despite the exposure to androgens during fetal life. However, further studies are needed to confirm and expand our findings.

##  Conflict of Interest

The authors declare that there are no conflict of interest. 
